# The relative and combined ability of stress hyperglycemia ratio and N-terminal pro-B-type natriuretic peptide to predict all-cause mortality in diabetic patients with multivessel coronary artery disease

**DOI:** 10.1186/s12933-024-02186-2

**Published:** 2024-03-11

**Authors:** Le Wang, Chen Wang, Jia-chun Lang, Rong-di Xu, Hong‑liang Cong, Jing‑xia Zhang, Yue‑cheng Hu, Ting-ting Li, Chun-wei Liu, Hua Yang, Wen‑yu Li

**Affiliations:** 1 Department of Cardiology, Tianjin Chest Hospital, Tianjin University, 261 Tai’erzhuang Road, Jinnan District, Tianjin, 300222 China; 2 Department of Cardiology, Chest Hospital, Tianjin University, 261 Tai’erzhuang Road, Jinnan District, Tianjin, 300222 China

**Keywords:** Stress hyperglycemia ratio, N-terminal pro-B-type natriuretic peptide, Long-term mortality, Diabetes, Multivessel disease

## Abstract

**Background:**

Stress hyperglycemia ratio (SHR) and N-terminal pro-B-type natriuretic peptide (NT-proBNP) are independently associated with increased mortality risk in diabetic patients with coronary artery disease (CAD). However, the role of these biomarkers in patients with diabetes and multivessel disease (MVD) remains unknown. The present study aimed to assess the relative and combined abilities of these biomarkers to predict all-cause mortality in patients with diabetes and MVD.

**Methods:**

This study included 1148 diabetic patients with MVD who underwent coronary angiography at Tianjin Chest Hospital between January 2016 and December 2016. The patients were divided into four groups according to their SHR (SHR-L and SHR-H) and NT-proBNP (NT-proBNP-L and NT-proBNP-H) levels. The primary outcome was all-cause mortality. Multivariate Cox regression analyses were performed to evaluate the association of SHR and NT-proBNP levels with all-cause mortality.

**Results:**

During a mean 4.2 year follow-up, 138 patients died. Multivariate analysis showed that SHR and NT-proBNP were strong independent predictors of all-cause mortality in diabetic patients with MVD (SHR: HR hazard ratio [2.171; 95%CI 1.566–3.008; P < 0.001; NT-proBNP: HR: 1.005; 95%CI 1.001–1.009; P = 0.009). Compared to patients in the first (SHR-L and NT-proBNP-L) group, patients in the fourth (SHR-H and NT-proBNP-H) group had the highest mortality risk (HR: 12.244; 95%CI 5.828–25.721; P < 0.001). The areas under the curve were 0.615(SHR) and 0.699(NT-proBNP) for all-cause mortality. Adding either marker to the original models significantly improved the C-statistic and integrated discrimination improvement values (all P < 0.05). Moreover, combining SHR and NT-proBNP levels into the original model provided maximal prognostic information.

**Conclusions:**

SHR and NT-proBNP independently and jointly predicted all-cause mortality in diabetic patients with MVD, suggesting that strategies to improve risk stratification in these patients should incorporate SHR and NT-porBNP into risk algorithms.

## Background

Patients with diabetes are prone to multivessel coronary artery disease (CAD) [1], which is associated with a higher risk of mortality than single-vessel disease [2]. Despite improvements in healthcare, patients with diabetes and multivessel disease (MVD) still have higher mortality rates than those without diabetes [3]. Therefore, risk stratification is crucial for identifying high-risk mortality to further improve the prognosis of patients with diabetes with MVD. However, the existing risk stratification model has only a moderate discrimination ability for individuals with diabetes and MVD [4]. Moreover, biomarkers are integral components of the risk stratification of patients with CAD [5–7]. Consequently, identifying biomarkers with strong prognostic value is of great importance for improving risk stratification in patients with diabetes with MVD.

Stress hyperglycemia, characterized by elevated blood glucose (ABG) levels upon admission, is an independent risk factor for mortality in patients with CAD [8, 9]. However, ABG has limitations in quantifying the degree of stress hyperglycemia because of the influence of the chronic glycemic state, particularly in patients with established DM [10]. The stress hyperglycemia ratio (SHR), calculated from ABG and glycosylated hemoglobin A1c (HbA1c), is a reliable marker of relative hyperglycemia [11]. Several studies have revealed an association between SHR and poor prognosis in patients with CAD [12–23]. Nevertheless, the association between SHR and long-term prognosis remains controversial [14, 18, 20, 23–26]. Moreover, the enrolled patients in previous studies were restricted to patients with myocardial infarction (MI) [18, 20, 23–25], acute coronary syndrome (ACS) [14], or chronic total occlusion (CTO) [26], but not those with MVD. A recent study showed that the SHR was significantly associated with the presence of MVD in patients with CAD [27]. To date, the effect of SHR on long-term mortality in patients with diabetes and MVD is unknown.

N-terminal pro-B-type natriuretic peptide (NT-proBNP), as a biomarker of myocardial stress, is a well-established diagnostic and prognostic marker for heart failure [28]. Numerous studies have demonstrated that increased NT-proBNP levels are associated with high all-cause mortality in diabetic patients with ischemic heart disease [29, 30]. Moreover, NT-proBNP has been proposed for risk assessment in patients with diabetes regardless of the presence of cardiovascular disease. Nevertheless, data regarding its role as a predictor of adverse outcomes in patients with MVD is limited [31]. NT-proBNP and its receptor not only regulate cardiovascular homeostasis, but are also involved in glucose metabolism [32]. Higher SHR levels significantly decreased left ventricular ejection fraction (LVEF) [33], and higher NT-proBNP levels strongly correlated with depressed systolic function and diastolic dysfunction [34]. However, little is known about the risk interaction between SHR and NT-proBNP levels in predicting all-cause mortality in patients with diabetes and CAD. Therefore, the purpose of the present study was to investigate the relative and combined prognostic values of SHR and NT-proBNP levels in patients with diabetes and MVD.

## Methods

### Study population

**Fig. 1:**
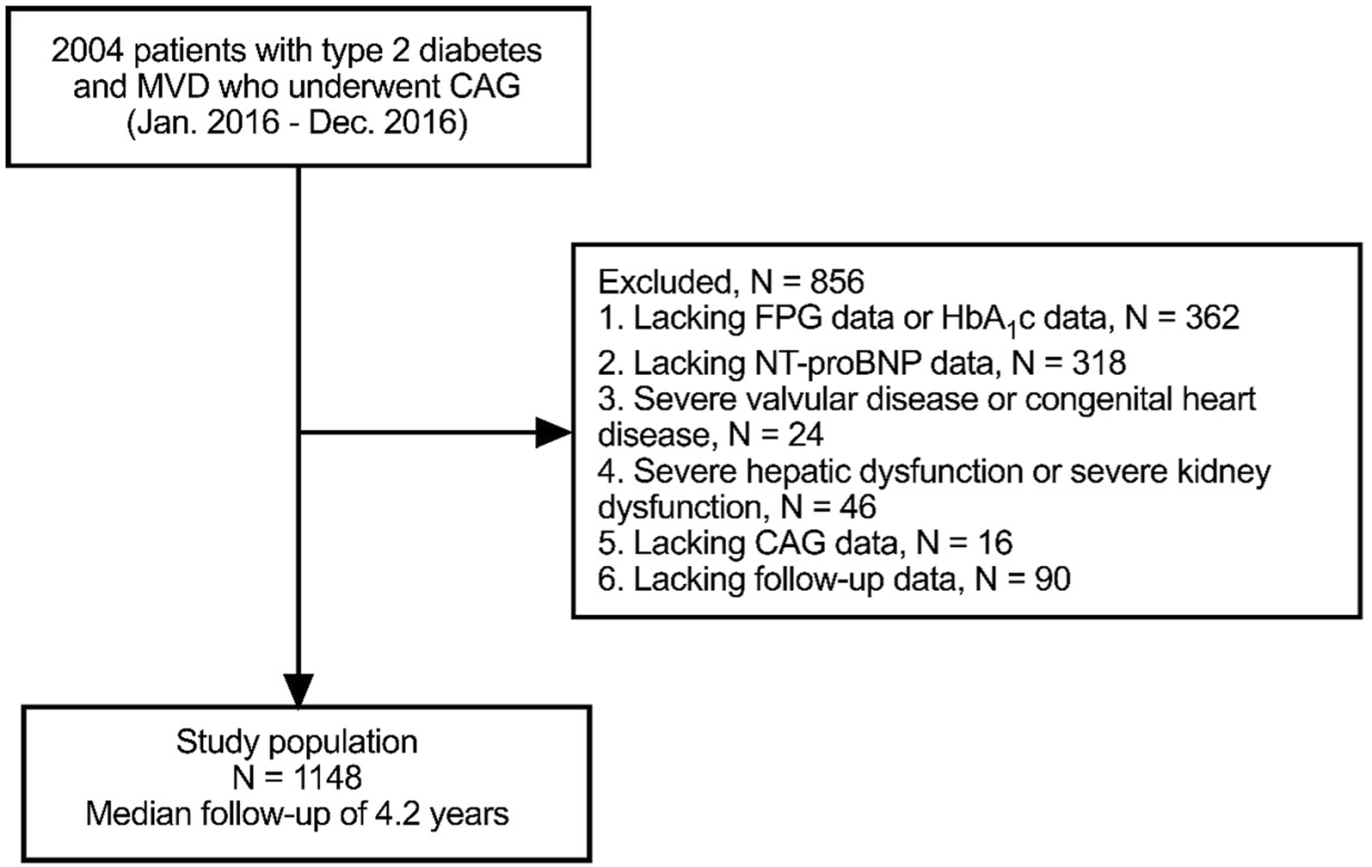
Patients flowchart

This was a retrospective, observational cohort study of MVD (defined as an angiographic diameter stenosis of ≥ 50% in at least two major epicardial coronary arteries, with or without involvement of the left main artery). A total of 2004 consecutive patients with type 2 diabetes and MVD who underwent coronary angiography (CAG) for chest pain between January 2016 and December 2016 at Tianjin Chest Hospital were included. Patients with type 2 diabetes were defined as those with a documented history of type 2 diabetes treated with medications or diets. CAD included stable angina pectoris (SAP) and ACS. The exclusion criteria were as follows: 1) those who had missing data on fasting plasma glucose (FPG) or glycosylated hemoglobin A1c (HbA1c) (n = 362); 2) those who had missing data on NT-pro BNP(n = 318);3) those who had severe valvular diseases or congenital heart disease (n = 24); 4) those who had a severe hepatic dysfunction (alanine transaminase level ≥ 5 times the upper reference limits) or severe kidney dysfunction (estimated glomerular filtration rate[eGFR] < 30 ml/min/1.73 m^2^) (n = 46); 5)those lacking CAG data(n = 16); 6) those lacking follow-up data(n = 90). Finally, 1148 patients were enrolled in this study (Fig. 1). All enrolled patients completed the clinical follow-up by telephone or outpatient visits between January 2020 and December 2020. The primary endpoint was all-cause mortality. Patients were divided into two groups according to the median level of fasting SHR (SHR-L group: < 0.79, n = 574; SHR-H group: ≥ 0.79, n = 574). Patients were divided into two groups according to the median level of NT-proBNP (NT-proBNP -L group: < 232.0 pg/ml, n = 574; NT-proBNP -H group: ≥ 232.0 pg/ml, n = 574). The patients were divided into four groups according to their fasting SHR and NT-proBNP levels (SHR-L + NT-proBNP-L group, n = 282; SHR-H + NT-proBNP-L group, n = 292; SHR-L + NT-proBNP-H group, n = 292; and SHR-H + NT-proBNP-H group, n = 282). This study was approved by the Ethics Committee of Tianjin Chest Hospital and was conducted in accordance with the Declaration of Helsinki. Considering the retrospective nature of this study, informed consent was not obtained from all patients.

### Data collection and definitions

All baseline clinical and laboratory data were collected from the electronic medical records by two trained investigators who were blinded to the purpose of the study. The clinical data included age, sex, weight, height, duration of diabetes, smoker proportion, history of hypertension, family history of CAD, history of myocardial infarction (MI), history of stroke, history of percutaneous coronary intervention (PCI), history of coronary artery bypass graft (CABG), clinical presentation, left ventricular ejection fraction (LVEF), three-vessel disease, left main(LM) disease, treatment strategies including medicine treatment, PCI and CABG, and medications (aspirin, clopidogrel/ticagrelor, β-blocker, angiotensin-converting enzyme inhibitor /angiotensin receptor blocker, statin, and insulin) at discharge. Laboratory findings included hemoglobin, first fasting plasma glucose (FPG), HbA1c, total cholesterol (TC), triglyceride (TG), low-density lipoprotein-C (LDL-C), high-density lipoprotein-C (HDL-C), high-sensitivity C-reactive protein (hs-CRP), NT-proBNP, and serum creatinine levels. Body mass index (BMI) was calculated as weight (kg)/ [height (m)] ^2^. The SHR was defined as [(first FPG (mmol/l))/(1.59 × HbA1c (%) -2.59)].

### Statistical analysis

Continuous variables are presented as mean ± standard deviation when normally distributed; otherwise, they are presented as medians with interquartile ranges. The differences between continuous variables were compared using Student’s t-test or the Mann–Whitney U test. Categorical variables are expressed as frequencies and percentages and analyzed using the chi-square test or Fisher’s exact test. The event-free survival rates among the groups were evaluated using the Kaplan–Meier method and log-rank tests. A multivariate Cox regression analysis with entry/stay criteria of 0.2/0.2 was used to determine the independent predictors of all-cause mortality. Possible factors included age, smoking, hypertension, previous MI, previous stroke, ACS, LVEF, hemoglobin, SHR, TC, TG, HDL-C, LDL-C, hs-CRP, NT-proBNP, serum creatinine, three-vessel disease, LM disease, aspirin, and statins. The association between individual biomarkers (categorical variables) and all-cause mortality was determined using multivariate Cox proportional hazard regression analyses. The optimal cutoff values of SHR and NT-proBNP for predicting all-cause mortality were determined using receiver operating characteristic (ROC) curves. Restricted cubic splines (RCSs) were used to assess the potential non-linear association between SHR, NT-proBNP levels, and all-cause mortality. To determine the discriminatory and reclassification ability of SHR and NT-proBNP over the original model (including age, smoking, ACS, LVEF, LM disease, and statins) for predicting all-cause mortality, C-statistics, integrated discrimination improvement (IDI), and category-free net reclassification improvement (NRI) were calculated. Statistical significance was defined as a two-sided *P*-value of < 0.05. Statistical analyses were performed using SPSS (version 20.0; IBM Corp., Armonk, NY, USA) and SAS software (version 9.1.3; Cary, NC, USA).

## Results

### Baseline characteristics of patients

The baseline patient characteristics are shown in Table 1. Over a mean 4.2 years of follow-up, 138 (12.0%) patients died. Of the 1148 patients in the present study, 57.0% were male, and the average age was 67.2 ± 6.9 years. Compared to survivors, non-survivors tended to be older (*P* < 0.001) and had a higher prevalence of smoking (*P* = 0.036), hypertension (*P* = 0.012), previous MI (*P* = 0.013), previous stroke (*P* = 0.015), and LM disease (*P* = 0.024). In addition, non-survivors had lower levels of LVEF (*P* < 0.001), haemoglobin (*P* = 0.007), TC(*P* = 0.041), and TG (*P* = 0.014), and higher levels of FPG (*P* < 0.001), SHR (*P* < 0.001), hs-CRP (*P* = 0.001), NT-proBNP (*P* < 0.001), and creatinine (*P* < 0.001). Meanwhile, the rate of statin use was lower at discharge in the non-survivors’ group compared in the survivor group (*P* = 0.018).

**Table 1:** Baseline characteristics of the study population

Clinical characteristics	Overall population	Non-survivors	Survivors	*P-* value
	(n = 1148)	(n = 138)	(n = 1010)	
Age, years	67.2 ± 6.9	70.9 ± 6.9	66.7 ± 6.8	< 0.001
Male	654 (57.0)	82 (59.4)	572 (56.6)	0.535
BMI, kg/m^2^	25.4 ± 2.8	25.7 ± 2.8	25.4 ± 2.8	0.321
Duration of diabetes, years	10.0(4.0–15.0)	9.5(4.8–14.3)	10.0 (4.0–15.0)	0.780
Smoker	471 (41.0)	68 (49.3)	403 (39.9)	0.036
Hypertension	934 (81.4)	123 (89.1)	811 (80.3)	0.012
Family history of CAD	118 (10.3)	14 (10.1)	104 (10.3)	0.956
Previous MI	176 (15.3)	31 (22.5)	145 (14.4)	0.013
Previous PCI	245 (21.3)	26 (18.8)	219 (21.6)	0.445
Previous CABG	68 (5.9)	10 (7.2)	58 (5.7)	0.483
Previous stroke	294 (25.6)	47 (34.1)	247 (24.5)	0.015
Clinical presentation				0.195
SAP	293(25.5)	29(21.0)	264 (26.1)	
ACS	855(74.5)	109(79.0)	746 (73.9)	
LVEF	58(52–62)	56(45–60)	58 (54–62)	< 0.001
Laboratory findings				
Hemoglobin, g/dl	131.0 ± 18.9	126.9 ± 19.7	131.5.1 ± 18.7	0.007
FPG, mmol/L	8.0 ± 3.1	9.4 ± 3.8	7.8 ± 2.9	< 0.001
HbA1c, %	7.7 ± 1.4	7.8 ± 1.4	7.7 ± 1.5	0.559
SHR	0.87 ± 0.40	1.02 ± 0.48	0.85 ± 0.38	< 0.001
TC, mmol/L	4.48 ± 1.20	4.29 ± 1.10	4.51 ± 1.21	0.041
TG, mmol/L	1.54(1.14–2.08)	1.35(1.06–1.90)	1.56(1.15–2.11)	0.014
HDL-C, mmol/L	1.03 ± 0.29	0.99 ± 0.27	1.04 ± 0.29	0.089
LDL-C, mmol/L	2.98 ± 1.00	2.84 ± 0.93	3.00 ± 1.02	0.084
hs-CRP, mg/L	2.16(0.82–5.96)	3.82(0.94–13.50)	2.03(0.81–5.38)	0.001
NT-proBNP, pg/ml Creatinine(umol/l)	232.0 (97.6–895.1)	839.8 (230.0–2414.5)	201.1 (88.9–712.5)	< 0.001
				< 0.001
Three-vessel disease	75.5 ± 27.4	86.3 ± 31.9	74.0 ± 26.4	0.058
Left main disease	856 (74.6)	112 (81.2)	744 (73.7)	0.024
Treatment	154 (13.4)	27 (19.6)	127 (12.6)	0.498
Medicine treatment				
PCI	271 (23.6)	37 (26.8)	234 (23.2)	
CABG	701 (61.1)	78 (56.5)	623 (61.7)	
Medications at discharge	176 (15.3)	23 (16.7)	153 (15.1)	
Aspirin				0.120
Clopidogrel/Ticagrelor	1117 (97.3)	131 (94.9)	986 (97.6)	0.896
β-blocker	1011 (88.1)	122 (88.4)	889 (88.0)	0.864
ACEI/ARB	781 (68.0)	93 (67.4)	688 (68.1)	0.625
Statin	660 (57.5)	82 (59.4)	578 (57.2)	0.018
Insulin	1100 (95.8)	127 (92.0)	973 (96.3)	0.689
	481 (41.9)	60 (43.5)	421 (41.7)	

*Data are expressed as mean* ± *SD, medians with interquartile ranges or percentage. BMI* body mass index*, CAD* coronary artery disease, *MI* myocardial infarction*, PCI* percutaneous coronary intervention*, CABG* coronary artery bypass graft*, SAP* stable angina pectoris*, ACS* acute coronary syndrome*, LVEF* left ventricle ejection fraction*, FPG* fasting plasm glucose*, HbA1c* Hemoglobin A1c, *SHR* stress hyperglycemia ratio, *TC* total cholesterol, *TG* triglycerides, *HDL-C* high-density lipoprotein cholesterol*, LDL-C low-density lipoprotein cholesterol, hs‐CRP high‐sensitivity C‐reactive protein, NT-proBNP N-terminal proB-type natriuretic peptide, ACEI* angiotensin II coenzyme inhibitor*, ARB* angiotensin II receptor blocker

There were no significant differences between the non-survivor group and survivor group in terms of sex ratio, BMI, duration of diabetes, family history of CAD, previous PCI, previous CABG, clinical presentation, HbA1c, HDL-C, LDL-C, three-vessel disease, treatment, or medications other than statins at discharge (all *P* > 0.05).

### Associations of SHR levels and all-cause mortality

The univariate and multivariate Cox proportional hazards regression analyses for all-cause mortality are shown in Table 2. In the univariate analysis, the variables associated with all-cause mortality were age, smoking, hypertension, previous MI, previous stroke, LVEF, hemoglobin, SHR, TC, hs-CRP, NT-proBNP, creatinine, LM disease, aspirin, and statins. When analyzed as continuous variables, multivariate Cox proportional hazards regression analysis showed that SHR (hazard ratio [HR], 2.171; 95% confidence interval [CI] 1.566–3.008; *P* < 0.001) and NT-proBNP level (HR, 1.005; 95%CI 1.001–1.009;* P* = 0.009) were independent predictors of mortality.

**Table 2:** Univariate and multivariate Cox regression analysis for all-cause mortality

Variables	Univariate			Multivariate		
	HR	95%CI	*P* value	HR	95%CI	*P* value
Age	1.084	1.059–1.110	< 0.001	1.089	1.060–1.119	< 0.001
Smoker	1.439	1.031–2.009	0.032	1.575	1.104–2.246	0.012
Hypertension	2.436	1.347–4.404	0.003			
Previous MI	1.649	1.105–2.459	0.014			
Previous stroke	1.563	1.099–2.223	0.013			
ACS	1.310	0.870–1.973	0.196	1.801	1.125–2.883	0.014
LVEF	0.953	0.939–0.968	< 0.001	0.962	0.943–0.982	< 0.001
Hemoglobin	0.990	0.984–0.997	0.006			
SHR	1.993	1.492–2.664	< 0.001	2.171	1.566–3.008	< 0.001
TC	0.866	0.754–0.995	0.042			
TG	0.849	0.698–1.032	0.100			
HDL-C	0.601	0.331–1.091	0.094			
LDL-C	0.865	0.731–1.022	0.088			
hs-CRP	1.007	1.003–1.011	0.011			
NT-proBNP (per 100 pg/ml)	1.011	1.008–1.013	< 0.001	1.005	1.001–1.009	0.009
Creatinine Three-vessel disease	1.007 1.507	1.004–1.010 0.983–2.309	< 0.001			
			0.060			
Left main disease	1.666	1.094–2.537	0.017	1.626	1.045–2.532	0.031
Aspirin	0.456	0.213–0.975	0.043			
Statin	0.452	0.244–0.837	0.012	0.494	0.248–0.982	0.044

*MI* myocardial infarction*, ACS* acute coronary syndrome*, LVEF* left ventricle ejection fraction*, SHR* stress hyperglycemia ratio, *TC* total cholesterol, *TG* triglycerides, *HDL-C* high-density lipoprotein cholesterol*, LDL-C* low-density lipoprotein cholesterol*, hs‐CRP* high‐sensitivity C‐reactive protein*, NT-proBNP* N-terminal proB-type natriuretic peptide*, HR* hazard ratio, *CI* confidential interval

**Fig. 2:**
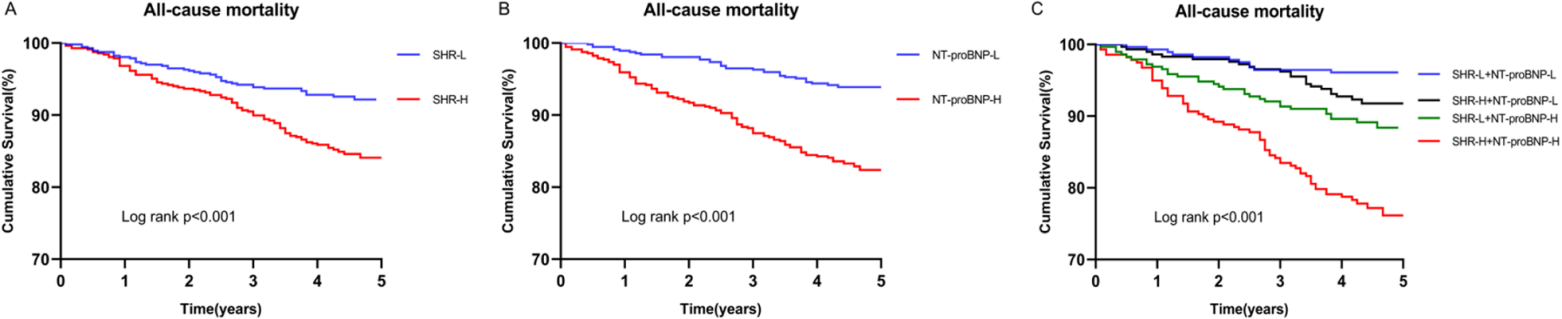
The event-free survival rate in SHR (A), NT-proBNP (B), and combined groups (C). SHR stress hyperglycemia ratio, NT-proBNP N-terminal pro-B-type natriuretic peptide

Patients were divided into two groups according to the median level of SHR (SHR-L group: < 0.79, n = 574; SHR-H group: ≥ 0.79, n = 574). As shown in Table 3, the all-cause mortality in the SHR-L and SHR-H groups were 8.0% and 16.0%, respectively (*P* < 0.001). As presented in Fig. 2A, Kaplan–Meier survival analysis showed that cumulative all-cause mortality increased with higher SHR levels (Log-rank* P* < 0.001). After adjusting for age, smoking status, hypertension, previous MI, previous stroke, ACS, LVEF, hemoglobin, TC, TG, HDL-C, LDL-C, hs-CRP, creatinine, three-vessel disease, left main disease, aspirin, and statins, multivariate Cox regression analysis showed that the SHR-H group had a higher risk of all-cause mortality than the SHR-L group (HR, 2.046; 95%CI 1.414–2.960; *P* < 0.001) (Table 3).

**Table 3:** Associations of SHR and NT-proBNP categories with all-cause mortality

Variable	Events, n/Total	Unadjusted model		Adjusted model	
		HR (95%CI)	*P* value	HR (95%CI)	*P* value
SHR			< 0.001		
Low	46/574(8.0)	Reference		Reference	
High	92/574(16.0)	2.072(1.454–2.952)		2.046(1.414–2.960)	< 0.001
NT-proBNP			< 0.001		
Low	36/574(6.3)	Reference		Reference	
High	102/574(17.7)	3.021(2.066–4.418)		5.739(3.365–9.789)	< 0.001
Combined categories			< 0.001		< 0.001
G1(SHR-L + NT-proBNP-L)	11/282(3.9)	References		References	
G2(SHR-H + NT-proBNP-L)	25/292(8.6)	2.225(1.095–4.522)	0.027	2.372(1.160–4.849)	0.018
G3(SHR-L + NT-proBNP-H)	35/292(12.0)	3.204(1.627–6.308)	0.001	6.587(3.012–14.406)	< 0.001
G4(SHR-H + NT-proBNP-H)	67/282(23.8)	6.753(3.569–12.778)	< 0.001	12.244(5.828–25.721)	< 0.001

*SHR* stress hyperglycemia, *NT-proBNP* N-terminal proB-type natriuretic peptide, *HR* hazard ratio; *CI* confidential intervals. Adjusted variables included age, smoker, hypertension, previous MI, previous stroke, ACS, LVEF, hemoglobin, TC, TG, HDL-C, LDL-C, hs-CRP, creatinine, three-vessel disease, left main disease, aspirin, and statin

**Fig. 3:**
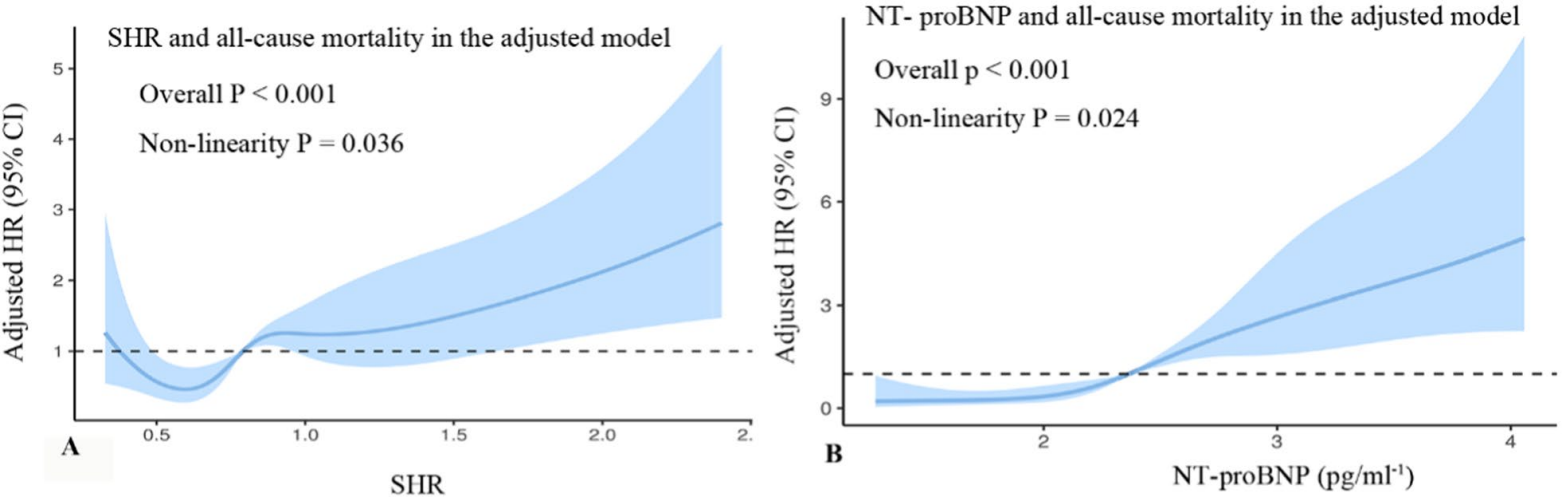
Restricted cubic spline curves for the association of SHR (A) and NT-proBNP (B) with the risk of all-cause mortality in the adjusted model. SHR stress hyperglycemia ratio, NT-proBNP N-terminal pro-B-type natriuretic peptide

**Fig. 4:**
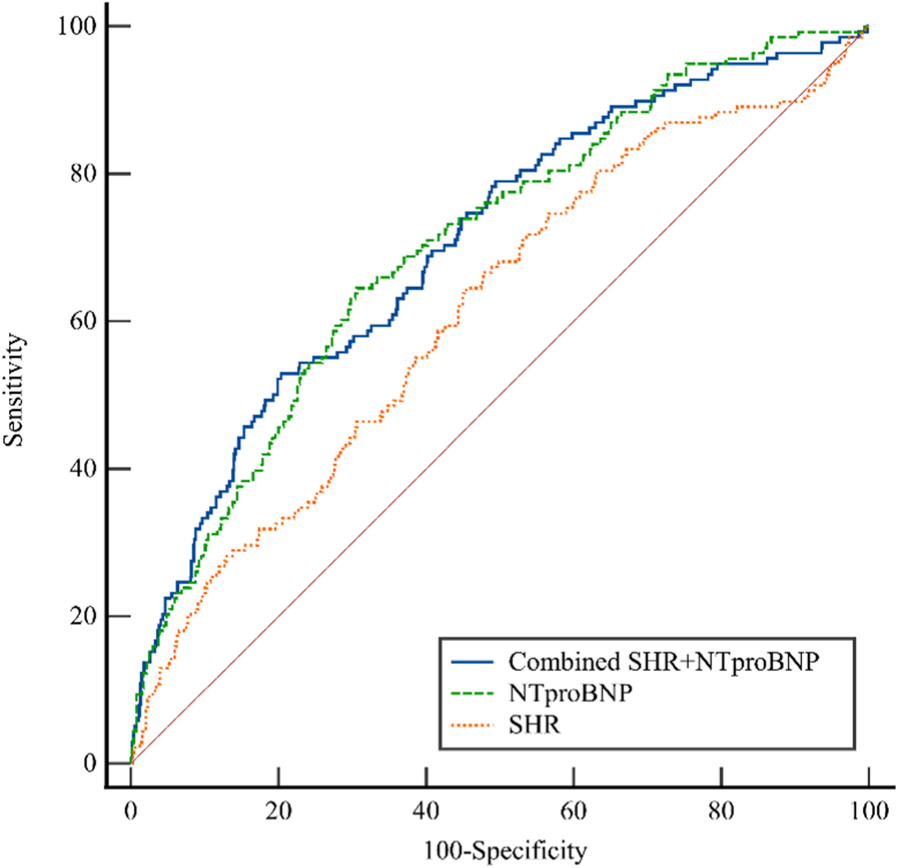
ROC curve for SHR, NT-proBNP and their combination in predicting all-cause mortality. Optimal cut-off: SHR:0.807; NT-proBNP: 511.8 pg/ml. AUC SHR: 0.614(0.585–0.642); AUC NT-proBNP:0.699(0.669–0.729); AUC combine:0.706(0.678–0.732). ROC receiver operating characteristic, AUC an area under the cure, SHR stress hyperglycemia ratio, NT-proBNP N-terminal pro-B-type natriuretic peptide

The RCS curves showed that SHR was positively and nonlinearly associated with the risk of all-cause mortality (*P* for nonlinearity = 0.036; Fig. 3A). For the predictive of SHR for all-cause mortality, ROC analysis showed that the optimal cutoff value of SHR for predicting all-cause mortality was 0.807(sensitivity: 64.49% and specificity: 54.55%), and the area under the curve (AUC) was 0.614(95%CI 0.585–0.642, *P* < 0.001) (Table 4, Fig. 4).

**Table 4:** ROC curve for SHR, NT-proBNP and their combination in predicting all-cause mortality

	AUC	Optimal cut-off value	Sensitivity %	Specificity %	95%CI	*P* value
SHR	0.614	0.807	64.49	54.55	0.585–0.642	< 0.001
NT-proBNP	0.699	511.8 pg/ml	64.49	69.41	0.669–0.729	< 0.001
SHR + NT-proBNP	0.706		52.90	79.60	0.678–0.732	< 0.001

*SHR* stress hyperglycemia, *NT-proBNP* N-terminal proB-type natriuretic peptide, *ROC* receiver operating characteristic, *AUC* an area under the cure, *CI* confidential intervals

### Associations of NT-proBNP levels and all-cause mortality

Similarly, patients were divided into two groups according to the median level of NT-proBNP (NT-proBNP -L group: < 232.0 pg/ml, n = 574; NT-proBNP -H group: ≥ 232.0 pg/ml, n = 574). The all-cause mortality in the NT-proBNP -L and NT-proBNP -H groups were 6.3% and 17.8%, respectively (*P* < 0.001) (Table 3). As shown in Fig. 2B, Kaplan–Meier survival analysis revealed that the cumulative all-cause mortality increased with higher NT-proBNP levels (Log-rank *P* < 0.001). When considered a categorical variable, the adjusted HR of higher NT-proBNP levels was 5.739 (95%CI 3.365–9.789; *P* < 0.001) (Table 3).

The RCS curves showed that NT-proBNP was positively and nonlinearly correlated with the risk of all-cause mortality (*P* for nonlinearity = 0.024; Fig. 3B). For the predictive of NT-proBNP for all-cause mortality, ROC analysis indicated that the optimal cutoff value of NT-proBNP for predicting all-cause mortality was 511.8 pg/ml (sensitivity: 64.49% and specificity: 69.41%), and AUC was 0.699(95%CI: 0.669–0.729, *P* < 0.001) (Table 4, Fig. 4).

### Inter-relationship of SHR, NT-proBNP levels and all-cause mortality

To evaluate the interaction between SHR, NT-proBNP, and all-cause mortality, patients were divided into four groups according to SHR and NT-proBNP levels [G1(SHR-L + NT-proBNP-L group, n = 282), G2(SHR-H + NT-proBNP-L group, n = 292); G3(SHR-L + NT-proBNP-H group, n = 292); G4(SHR-H + NT-proBNP-H group, n = 282]). The all-cause mortality in the four groups were 3.9%, 8.6%, 12.0%, and 23.8%, respectively (*P* < 0.001) (Table 3). Compared to G1 group, groups G2, G3, and G4 had 2.225-fold, 3.204-fold, and 6.753-fold higher risks of all-cause mortality, respectively. After adjusting for potential confounding factors, the G2 group, G3 group and G4 group had 2.372-fold, 6.587-fold, and 12.244-fold higher risks of all-cause mortality [HR (95%CI) 2.372(1.160–4.849), *P* = 0.018; 6.587(3.012–14.406),* P* < 0.001; 12.244(5.828–25.721),* P* < 0.001, respectively]. (Table 3). As shown in Fig. 2C, Kaplan–Meier survival analysis showed that the cumulative all-cause mortality in G4 group was the highest among the four groups (Log-rank *P* < 0.001).

### Incremental value of SHR, NT-proBNP over original model for all-cause mortality

As shown in Table 5, the C-statistic of the original model including age, smoker, ACS, LVEF, LM disease and statin was 0.735(95%CI 0.691–0.770) for all-cause mortality. The addition of SHR to the original model improved the prediction of all-cause mortality in terms of the C-statistic (0.758; 95%CI 0.716–0.801; *P* = 0.017), the NRI (0.296;95%CI 0.120–0.473; *P* = 0.001) and the IDI (0.021;95%CI 0.007–0.034; *P* = 0.003), respectively.

**Table 5:** Additional predictive value provided by SHR and NT-proBNP for predicting all-cause mortality

	C-Statistic	*P* value	NRI (95%CI)	*P* value	IDI (95%CI)	*P* value
Original model	0.735 (0.691–0.779)	References				
Original model + SHR	0.758 (0.716–0.801)	0.017	0.296 (0.120–0.473)	0.001	0.021 (0.007–0.034)	0.003
Original model + NT-proBNP	0.750 (0.706–0.793)	0.009	− 0.010 (− 0.185–0.165)	0.911	0.015 (0.002–0.028)	0.027
Original model + SHR + NT-proBNP	0.772 (0.731–0.813)	< 0.001	0.340 (0.163–0.517)	< 0.001	0.034 (0.015–0.053)	< 0.001

*SHR* stress hyperglycemia, *NT-proBNP* N-terminal proB-type natriuretic peptide, *NRI* net reclassification improvement, *IDI* integrated discrimination improvement, *CI* confidential intervals. Original model included age, smoker, ACS, LVEF, left main disease and statin

Adding NT-proBNP to the original model improved the prediction of all-cause mortality in terms of the C-statistic (0.750; 95%CI 0.706–0.793; *P* = 0.009) and IDI (0.015;95%CI 0.002–0.028; *P* = 0.027), but not the NRI (− 0.010;95%CI − 0.185− 0.165; *P* = 0.911) (Table 5). The combination of SHR and NT-proBNP levels with the original model provided better prognostic information in terms of the C-statistic (0.772; 95%CI 0.731–0.813; *P* < 0.001), NRI (0.340;95%CI 0.163–0.517; *P* < 0.001), and IDI (0.034;95%CI 0.015–0.053; *P* < 0.001) (Table 5).

As shown in Table 6, adding SHR to the original model plus NT-proBNP significantly improved the C-statistics, NRI, and IDI (all *P* < 0.05). However, the combination of NT-proBNP to the original model plus SHR only significantly improved the C-statistic and IDI (all *P* < 0.05), but not the NRI (*P* > 0.05).

**Table 6:** Additional predictive value after the addition of SHR or NT-proBNP to original model containing the other marker

	C-Statistic	P value	NRI (95%CI)	P value	IDI (95%CI)	P value
Original model + NT-proBNP SHR + original model + NT-proBNP	0.750 (0.706–0.793) 0.772 (0.731–0.813)	0.026	0.289 (0.112–0.465)	0.002	0.019 (0.006–0.032)	0.003
Original model + SHR NT-proBNP + original model + SHR	0.758 (0.716–0.801) 0.772 (0.731–0.813)	0.009	0.039 (− 0.137–0.215)	0.666	0.013 (0.001–0.026)	0.040

*SHR* stress hyperglycemia, *NT-proBNP* N-terminal proB-type natriuretic peptide, *NRI* net reclassification improvement, *IDI* integrated discrimination improvement, *CI* confidential intervals. Original model included age, smoker, ACS, *LVEF* left main disease and statin

## Discussion

This is the first study to focus on diabetic patients with MVD and investigate the association of SHR and NT-proBNP with the prediction of all-cause mortality. The major findings of this study are as follows: First, SHR and NT-proBNP levels were positively associated with an increased risk of all-cause mortality. The SHR was an independent predictor of all-cause mortality when added to a multivariate model including NT-proBNP levels. Second, when the SHR and NT-proBNP categories were combined, the combination significantly enhanced the predictive value of these markers by increasing the risk of mortality. Patients in the SHR-H and NT-proBNP-H groups had a 12.244-fold increased risk of mortality compared to those in the fasting SHR-L and NT-proBNP-L groups. Third, the addition of each biomarker to the established model significantly improved the discriminatory and reclassification abilities for all-cause mortality prediction. The combination of SHR and NT-proBNP levels in the model provided maximal prognostic information. Notably, the SHR and NT-proBNP levels provided prognostic information that was incremental to each other. To the best of our knowledge, this is the first study to confirm the prognostic value of SHR in patients with diabetes and MVD. Most importantly, combining SHR and NT-proBNP is of great importance for improving risk stratification in patients with diabetes and MVD.

SHR, as a new biomarker, reflects the true acute hyperglycemia status and may more accurately identify stress hyperglycemia by attenuating the impact of background glycemic status [11]. SHR have been demonstrated to be better prognostic predictors of critical diseases than ABG [15, 23, 35, 36]. Increasing evidence suggests that a higher SHR is significantly associated with a higher risk of poor short- and long-term prognoses in patients with ACS. Moreover, similar findings have been observed in patients with chronic coronary syndrome [17] and CTO lesions [26]. These results suggest that SHR may be a useful predictive marker of poor prognosis in patients with or without stress conditions. SHR have been demonstrated to be associated with the risk of MVD in diabetic patients with CAD, but not in patients with normal glucose metabolism [27]. Compared to patients with single-vessel CAD, those with MVD have a worse long-term prognosis. However, the existing model has only a modest discrimination ability for mortality prediction in patients with MVD, suggesting that the increased risk of death in patients with diabetes cannot be simply predicted using conventional risk factors [4]. Therefore, evaluating the potential role of the SHR as a prognostic biomarker may have great clinical significance for risk stratification in patients with diabetes and MVD.

However, it is unclear whether SHR are associated with the long-term prognosis of patients with diabetes and CAD. In 2021, Sia et al. conducted a national registry-based study of 9946 patients with acute myocardial infarction (AMI) and found that SHR was independently associated with 1 year all-cause mortality in diabetic and non-diabetic patients with AMI [23]. Luo et al. demonstrated in a study of 2089 AMI patients with a median follow-up of 2.7 years that SHR was an independent predictor of all-cause mortality, irrespective of diabetic status [18]. Similarly, Zeng et al. analyzed the data of 7662 patients with ACS from a national perspective cohort and found that higher SHR levels were independently correlated with an increased risk of all-cause death in patients with and without diabetes [14]. In addition, according to a study of 2311 AMI patients with a median follow-up time of 6.5 years, Schmitz et al. discovered that the association between SHR and 5 year all-cause mortality was significant only in diabetic patients and not in patients without diabetes [20]. In contrast, several studies have not demonstrated a significant association between the SHR and the long-term prognosis of patients with diabetes and CAD. In a study of 6,287 STEMI patients with a follow-up 5 years, Kojima et al. (2020) reported that the highest SHR quartile was significantly associated with all-cause death and heart failure admission in non-diabetic patients but not in diabetic patients [25]. Data from 4337 AMI patients from American and Chinese cohorts with a maximum follow-up time of more than 14 years showed that elevated SHR was significantly associated with 1 year and long-term all-cause mortality in patients without diabetes, but not in those with DM [24]. The potential reasons for these discrepancies may be attributed to variations in patient characteristics, disease severity, and follow-up among the studies. Moreover, the prognostic value of SHR has not been evaluated in patients with diabetes and confirmed MVD.

Compared with previous studies, the present study included higher-risk diabetic patients with ACS and SAP, and the mean follow-up period was 4.2 years. This study demonstrated that higher SHR, whether as a continuous or categorical variable, was associated with a higher risk of all-cause mortality. This relationship persisted after adjusting for traditional cardiovascular risk factors, clinical presentation, cardiac function, laboratory parameters, coronary artery disease severity, and medication. These findings highlight that SHR is a strong independent predictor of all-cause mortality in patients with diabetes with MVD. Furthermore, RCS curves showed a positive non-linear association between SHR and all-cause mortality, which was inconsistent with the study conducted by Luo et al. [18]. This difference may be attributable to the different clinical characteristics of the patients. The present study identified the optimal cutoff value of SHR and found that the AUC for predicting mortality was poor, suggesting that SHR alone cannot provide sufficient prognostic information for diabetic patients with MVD. However, adding SHR to the established risk factors for all-cause mortality provided additional prognostic information by improving C-statistics and IDI. Therefore, in diabetic patients with MVD, SHR was not only an independent prognostic biomarker of all-cause mortality, but also might enhance risk discrimination when combined with the established risk factors for all-cause mortality, suggesting that routine SHR calculations may be amenable for refining risk stratification in this high-risk group.

NT-proBNP is an established diagnostic and prognostic tool for patients with chronic cardiovascular conditions, including heart failure and CAD. The independent association of NT-proBNP and adverse prognosis has also been previously demonstrated in diabetic patients with or without CAD [29, 30, 37, 38]. However, recent guidelines do not recommend routine NT-proBNP measurements for risk prediction in diabetic patients [39]. Only one previous study investigated the prognostic utility of NT-proBNP in patients with diabetes and MVD undergoing coronary revascularization. Wang et al. found that higher procedural NT-proBNP levels were associated with all-cause death and that adding NT-proBNP to the SYNTAX II score significantly improved risk prediction of all-cause mortality [31]. Unlike previous studies, the present study included patients with a history of revascularization and those treated with medication. The present study extended previous findings, confirming that NT-proBNP is a strong predictor of all-cause mortality, and that the combination of NT-proBNP with clinical factors may accurately discriminate the risk of mortality. Moreover, this study also revealed a non-linear association between NT-proBNP and all-cause mortality, and the AUC and optimal cutoff of NT-proBNP for predicting all-cause mortality in diabetic patients with MVD. Based on these findings, NT-proBNP may be regarded as a useful biomarker for the risk stratification of patients with diabetes and MVD.

Currently, evidence regarding the relationship between SHR, NT-proBNP levels, and mortality is scarce. Although higher SHR levels may reduce LVEF and higher NT-proBNP levels may reflect depressed systolic function, SHR and NT-proBNP independently predicted all-cause mortality in the present study, indicating that SHR and NT-proBNP cannot be substituted for each other in prognostic evaluation. Therefore, an increased SHR or NT-proBNP level in diabetic patients with MVD should be considered a prognostic biomarker for higher mortality risk. Furthermore, the SHR and NT-proBNP are viable tools for risk stratification in patients with diabetes and MVD. However, SHR and NT-proBNP levels had only modest predictive values for mortality. The AUC of NT-proBNP for predicting mortality risk was larger than that of SHR, indicating that the predictive value of NT-proBNP was better than that of SHR in diabetic patients with MVD. Nevertheless, this study does not suggest that NT-proBNP should replace SHR in risk stratification. When these two biomarkers were evaluated in the context of a baseline clinical model including age, smoking status, ACS, LVEF, left main disease, and statin use, the C-statistics for the prediction of all-cause mortality were similar. This result may be partly attributed to the fact that there is a certain degree of overlap between the two biomarkers and is an established predictor of all-cause mortality.

Given the potentially close relationship between SHR and NT-proBNP levels, this study divided the study population into four groups according to the median levels of each biomarker. The results showed that patients with dual elevations of these two markers had a significantly higher risk of mortality than those with low SHR and NT-proBNP levels. Moreover, adding the two biomarkers to the established model for all-cause mortality offered significant incremental value compared to the addition of any biomarker (SHR or NT-proBNP) in terms of C-statistics and IDI. This finding emphasizes the advantage of the combined detection of these two biomarkers for accurately predicting mortality risk. To the best of our knowledge, the present study is the first to explore the combined prognostic value of SHR and NT-proBNP in patients with diabetes and to demonstrate the additive effect of these two biomarkers. However, the mechanisms underlying the independent and joint associations of SHR and NT-proBNP levels with all-cause mortality in patients with diabetes and MVD remain unclear. The association between SHR, NT-proBNP levels, and mortality risk cannot be completely attributed to declining cardiac function. In fact, SHR may reflect glucose metabolism disorders and disease-related stress, and NT-proBNP levels may reflect responses to increased stress on cardiomyocytes and volume overload. The level of NT-proBNP is strongly related to myocardial ischemia burden [40] and diabetes-related complications [41] in diabetic patients with CAD. These differences may partially explain why these two biomarkers provide incremental prognostic information. Further studies are needed to elucidate the exact mechanisms in diabetic patients with MVD. These findings support the hypothesis that the combined use of different biomarkers reflecting different pathophysiological mechanisms is more informative for risk prediction. It should be acknowledged that measurement of FPG, HbA1c, and NT-proBNP levels is feasible using blood tests in clinical practice. Thus, simultaneous assessment of SHR and NT-proBNP levels should be considered when stratifying diabetic patients with MVD for future mortality risk.

## Study limitations

This study has several limitations. First, as this was a single-center retrospective study, residual unmeasured confounders could not be excluded, despite comprehensive adjustment for important cardiovascular risk factors. The lack of information on diabetic complications and antidiabetic drugs makes it difficult to determine their impact on the association between SHR, NT-proBNP, and mortality. This aspect should be considered in future studies. Second, the study population only included diabetic patients with MVD. Owing to the limited sample size and relatively low incidence of events, a subgroup analysis was not conducted. However, these findings need to be validated in different populations. Third, FPG, HbA1c, and NT-proBNP levels were measured only at baseline. Information on these biomarkers was not collected after discharge. Therefore, the prognostic value of dynamic changes in SHR and NT-proBNP levels for all-cause mortality warrants further investigation. Fourth, the use of sodium glucose co-transporter 2 inhibitors (SGLT2-i) and glucagon-like peptide 1 receptor agonist (GLP-1RA) therapy may reduce the risk of mortality and HF hospitalizations [42, 43]. However, SGLT2-i and GLP-1RA were not used in our clinical practice in 2016. Therefore, information regarding the use of these drugs is unavailable. The lack of information on the use of SGLT2-i and GLP-1RA may have exaggerated the results of this study. Finally, the follow-up information mainly included survival data. Follow-up information was collected via telephone calls or outpatient visits. The exact cause of death for patients who died outside our hospital could not be determined. Many studies have investigated the association between SHR and all-cause mortality [24, 44, 45]. Therefore, cardiovascular mortality, nonfatal MI, and nonfatal stroke were not included in this study. Future prospective studies are required to evaluate the association between SHR and these endpoints. Despite these limitations, the present study is the first to investigate the independent and joint associations of SHR and NT-proBNP levels with all-cause mortality in patients with diabetes and MVD.

## Conclusion

This is the first study to demonstrate that elevated SHR and NT-proBNP levels are independent predictors of all-cause mortality in patients with diabetes and MVD. There were complementary effects between SHR and NT-proBNP in predicting all-cause mortality, and adding them to the basic model may significantly enhance predictive accuracy. These novel findings suggest that strategies to improve risk stratification in patients with diabetes and MVD should incorporate the SHR and NT-porBNP into risk algorithms.

## Data Availability

The raw data supporting the conclusions of the current study are available from the corresponding author on reasonable request.

## Abbreviations

SHR: Stress hyperglycemia ratio
NT-proBNP: N-terminal pro-B-type natriuretic peptide
CAD: Coronary artery disease
MVD: Multivessel disease
HR: Hazard ratio
ABG: Admission blood glucose
HbA1c: Glycosylated hemoglobin A1c
MI: Myocardial infarction
ACS: Acute coronary syndrome
CTO: Chronic total occlusion
LVEF: Left ventricular ejection fraction
CAG: Coronary angiography
eGFR: Estimated glomerular filtration rate
PCI: Percutaneous coronary intervention
CABG: Coronary artery bypass graft
LM: Left main
ACEI: Angiotensin-converting enzyme inhibitor
ARB: Angiotensin receptor blocker
FPG: Fasting plasm glucose
TC: Total cholesterol
TG: Triglycerides
LDL-C: Low-density lipoprotein-C
HDL-C: High-density lipoprotein-C
hs-CRP: High-sensitivity C-reactive protein
BMI: Body mass index
ROC: Receiver operating characteristic
RCS: Restricted cubic spline
IDI: Integrated discrimination improvement
NRI: Net reclassification improvement
AUC: Area under the curve
AMI: Acute myocardial infarction (AMI)
SGLT2-i: Sodium glucose co-transporter 2 inhibitors
GLP-1RA: Glucagon-like peptide 1 receptor agonists
